# Spatial and Single‐Cell Transcriptomics Unraveled Spatial Evolution of Papillary Thyroid Cancer

**DOI:** 10.1002/advs.202404491

**Published:** 2024-11-14

**Authors:** Guangzhe Zheng, Shaobo Chen, Wanqi Ma, Quanshu Wang, Li Sun, Changwen Zhang, Ge Chen, Shuping Zhang, Shuguang Chen

**Affiliations:** ^1^ Medical Science and Technology Innovation Center Shandong First Medical University & Shandong Academy of Medical Sciences Jinan Shandong 250117 China; ^2^ Department of General Surgery Peking Union Medical College Hospital Chinese Academy of Medical Sciences & Peking Union Medical College Beijing 100032 China; ^3^ Biomedical Sciences College & Shandong Medicinal Biotechnology Centre Shandong First Medical University & Shandong Academy of Medical Sciences Jinan Shandong 250117 China; ^4^ The First Affiliated Hospital of Shandong First Medical University Jinan Shandong 250014 China; ^5^ Department of Urology Tianjin Institute of Urology The Second Hospital of Tianjin Medical University Tianjin 300211 China; ^6^ School of Public Health Shandong First Medical University & Shandong Academy of Medical Sciences Jinan Shandong 250117 China

**Keywords:** intratumor spatial heterogeneity, papillary thyroid cancer, spatial evolution, spatially‐resolved transcriptomics

## Abstract

Recurrence and metastasis are the major issues for papillary thyroid cancer (PTC). Current morphological and molecular classification systems are not satisfied for PTC diagnosis due to lacking variant‐specific morphological criteria and high signal‐to‐noise in mutation‐based diagnosis, respectively. Importantly, intratumor heterogeneity is largely lost in current molecular classification system, which can be resolved by single cell RNA sequencing (scRNA‐seq). However, scRNA‐seq loses spatial information and morphological features. Herein, scRNA‐seq is integrated and spatially‐resolved transcriptomics (SRT) to elaborate the mechanisms underlying the spatial heterogeneity, malignancy and metastasis of PTCs by associating transcriptome and local morphology. This results demonstrated that PTC cells evolved with multiple routes, driven by the enhanced aerobic metabolism and the suppressed mRNA translation and protein synthesis and the involvement of cell–cell interaction. Two curated malignant and metastatic footprints can discriminate PTC cells from normal thyrocytes. Ferroptosis resistance contributed to PTC evolution. This results will advance the knowledge of intratumor spatial heterogeneity and evolution of PTCs at spatial and single‐cell levels, and propose better diagnostic strategy.

## Graphical abstract

Integrated analysis of scRNA‐seq and SRT, by associating transcriptome and local morphology, reveals that enhanced aerobic metabolism, suppressed mRNA translation and protein synthesis, and cell–cell interactions collectively drive the evolution of PTC cells. This finding further elucidates the mechanisms of spatial heterogeneity, malignancy, and metastasis of PTC.

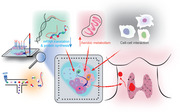

## 1 Introduction

Thyroid cancer is one of the most common cancers, and papillary thyroid cancer (PTC) is the predominant subtype of thyroid cancer with approximate 85% occupancy.^[^ ^1^ ^]^ PTCs are generally treated as an indolent and curable cancer type with a 10‐year survival rate >90% after conventional treatments. However, two key issues, recurrence and metastasis, still hamper the clinical outcome and cause the increased mortality for patients with PTCs. PTCs possess more than fifteen distinct histological variants defined by qualitative or semiquantitative morphological criteria. However, these criteria are not specific to any variant, leading to inherent difficulties in PTC variant diagnosis based on the histological features.^[^ ^3^ ^]^

From a genetic alteration of view, B‐Raf proto‐oncogene, serine/threonine kinase (BRAF) mutation, Rat Sarcoma (RAS) mutation and rearranged during transfection/papillary thyroid carcinoma chromosomal rearrangements are presented in PTCs.^[^ ^4^ ^]^ Nonetheless, PTCs possess a lower overall mutation rate compared to other cancer types,^[^ ^5^ ^]^ leading to an increase of signal‐to‐noise ratio in mutation‐based classification and diagnose of PTCs.^[^ ^6^ ^]^ According to the abnormal molecular signaling profiles at bulk level, PTCs can be identified as BRAF‐like or RAS‐like subtypes associated with distinct biological features, dedifferentiation and aggressiveness.^[^ ^4^ ^]^ For example, constitutively activated MAPK pathway is frequently observed in BRAF‐like PTCs compared to the constitutive activation of both MAPK and PI3K pathways in RAS‐like PTCs.^[^ ^4^ ^]^ Nuclear factor kappa‐light‐chain‐enhancer of activated B cells pathway is also frequently activated to promote cell proliferation and inhibit cell apoptosis in PTCs, such as in BRAF^V600E^ PTCs.^[^ ^4^, ^7^ ^]^ Compared to the morphological diagnosis, molecular diagnosis is more effective benefiting from the quantitative scores of molecular phenotypes and markers.^[^ ^6^ ^]^

However, intratumor heterogeneity is largely lost in the current morphological and molecular classification systems for thyroid cancers. The stromal cells in the microenvironment of thyroid cancers, including PTCs, consist of lymphocytes, macrophages, fibroblasts, endothelial cells (ECs) and other cells. The composition and proportions of these stromal cells vary across thyroid cancers, patients and even within patients, leading to a caution to reliably diagnose the malignancy and progression of PTCs using the specific markers identified in bulk tumor tissue analyses.^[^ ^6^ ^]^

Emerging technologies, mostly single cell RNA sequencing (scRNA‐seq), have been applied to provide unbiased identification of cell subtypes and cell states in PTCs by targeting the heterogeneities of both cancer cells and TME at single‐cell level.^[^ ^8^ ^]^ However, spatial information is discarded in these scRNA‐seq analyses due to tissue digestion and cell dissociation. Spatially‐resolved transcriptomics (SRT) is a new technology developed to target this issue.^[^ ^9^ ^]^ However, different from the single‐cell resolution of scRNA‐seq, SRT is currently performed at spot‐level. Each SRT spot is a mixture of multiple cells or even multiple types of cells, leading to a mixed expression profile within each spot. Thus, integration of scRNA‐seq and SRT is currently necessary to provide both single‐cell transcriptome and spatial distribution. For example, anchor‐based projection of the cell subtypes and gene markers from scRNA‐seq onto SRT slide with hematoxylin‐eosin (H&E) image would reveal the association of transcriptome and local morphology to elaborate the mechanisms underlying the morphological characteristics and the recurrence and metastasis of cancers.^[^ ^6^ ^]^

In the current study, we employed the SRT technique, which offers a resolution of 55 µm over ≈5000 spots, to comprehensively reveal the spatial features of PTCs with or without metastasis, including intratumoral and spatial heterogeneity, and spatial evolutionary routes. Our results revealed high spatial heterogeneity, multiple evolutionary routes and gene footprints inside the primary PTC tissue with lymph metastasis.

## 2 Results

### 2.1 Greater Spatial Heterogeneity in the Primary PTC Tissue with Metastasis

**Figure 1:**
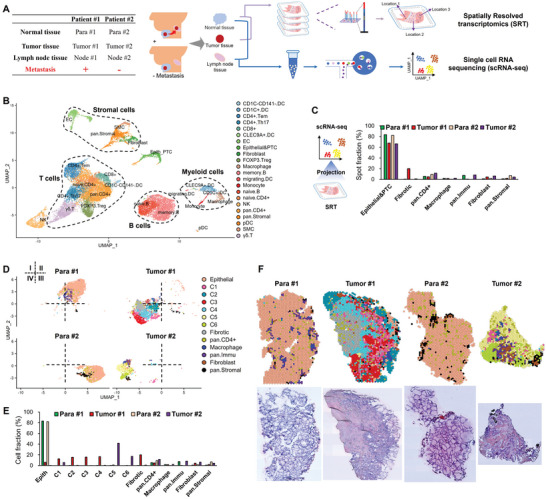
Overview of scRNA‐seq and SRT analysis and spatial heterogeneity in PTCs. A) Workflow of scRNA‐seq and SRT analyses on PTC samples. B) Dimension reduction by Uniform Manifold Approximation and Projection (UMAP) visualization of cell clusters annotated in scRNA‐seq. C) Projection of cell clusters of scRNA‐seq on the spots of SRT analysis, and the fraction of spots annotated. D–F) UMAP D), spatial visualization F) and fractions E) of spot clusters classified in SRT analysis. Para #1 and #2, para‐carcinoma tissues from patient #1 and #2, respectively; Tumor #1 and #2, tumor tissues from patient #1 and #2, respectively; Node #1 and #2, matched lymph node tissues from patient #1 and #2, respectively.

To fully characterize the cell heterogeneity of PTC tissues, six samples were collected from one PTC patient with lymph node metastasis (denoted as Patient#1), and another PTC patient without metastasis (denoted as Patient#2); both of these two patients had BRAF^V600E^ mutation (**Figure** **1**A and Table S1, Supporting Information). For each patient, primary tumor tissues (denoted as Tumor#1 and #2, respectively), matched para‐carcinoma tissues (denoted as Para#1 and #2, respectively) and lymph node tissues (denoted as Para#1 and #2, respectively) were collected and subjected to scRNA‐seq analysis. Tumor and para‐carcinoma tissues from both two patients were also subjected to SRT analysis (Figure 1A).

A total of 21060 cells and 17601 genes passed quality control in scRNA‐seq (Figure S1A, Supporting Information), and further annotated into 22 major cell clusters using cell markers (Figure 1B). These cell clusters belonged to five categories, T cells, B cells, myeloid cells, stromal cells, and a mixed cluster of epithelial and PTC cells (denoted as Epithelial & PTC) (Figure 1B). Furthermore, 0.9% of the qualified cells were PTC cells in Node #1 tissue but not in Node#2 tissue, confirming the lymph metastasis of PTC cells in Patient#1 but not in Patient#2 (Figure S1B, Supporting Information). High infiltration of immune cells was found in both tumor and para‐carcinoma tissues (Figure S1C, Supporting Information), consistent with previous reports of high immune cell infiltration in PTCs.^[^ ^8^, ^10^ ^]^ By analyzing the composition of immune cells, B cells, natural killer cells and stromal cells were increased while T cells and myeloid cells were decreased in Tumor#1 tissue compared to Tumor #2 tissue (Figure S1C, Supporting Information).

In SRT analysis, 6862 spots (1‐10 cells per spot) and 20582 in situ expressed genes were totally obtained (Figure S1D, Supporting Information). After the projection of annotated cell clusters from scRNA‐seq onto the spot clusters from SRT combining with gene marker annotation strategy, 66.3%‐83.5% of the spots were classified as epithelial or PTC spots (Figure 1C). The rest spots were classified as fibrotic spots (located in fibrotic area), pan.CD4+ T spots, macrophages, pan.Immu spots, fibroblasts and pan.Stromal spots (Figure 1C; Figure S1E, Supporting Information). Total read counts and gene expression intensity per spot were highest in epithelial and PTC spots, and lowest in fibrotic spots (Figure S2A, Supporting Information), consistent with the report of diverse spatial transcriptomics of PTCs by Saiselet and colleagues.^[^ ^11^ ^]^ Tumor #1 tissue possessed the greatest spatial heterogeneity as demonstrated by the results of global spot diversities (Figure S2B, Supporting Information), revealing a greater spatial heterogeneity in the primary tissue of patient #1 with metastasis.

According to the histological characteristics and unsupervised clustering, PTC spots were divided into six major morphological spot clusters (C1‐C6), in which C2‐4 exclusively existed in Tumor #1 tissue while C5‐6 exclusively existed in Tumor #2 tissue (Figure 1D,E). PTC spots exhibited an interlaced distribution pattern in both Tumor #1 and #2 tissues, particularly in Tumor #1 tissue (Figure 1F). The papillae architecture with a progressively disappeared follicular structure is the classic characteristic of PTCs, in which PTC cells are organized around a fibrous structure that is sometimes vascularized. PTC cells possess enlarged and ground glass nuclei, an arguable hallmark of PTCs.^[^ ^6^ ^]^ Thus, C3 from Tumor #1 tissue and C6 from Tumor #2 tissue possessed the classic PTC features of papillae fibrotic cores with malignant epithelial cells on the periphery, suggesting that cells in C3 and C6 spots were the classic malignant PTC cells (Figure 1F).

Collectively, these results demonstrated that integration of scRNA‐seq and SRT data can reveal the spatial heterogeneity, which was enhanced in PTCs with metastasis.

### 2.2 Multiple PTC Evolution Routes in the Primary Tissues with Metastasis

**Figure 2:**
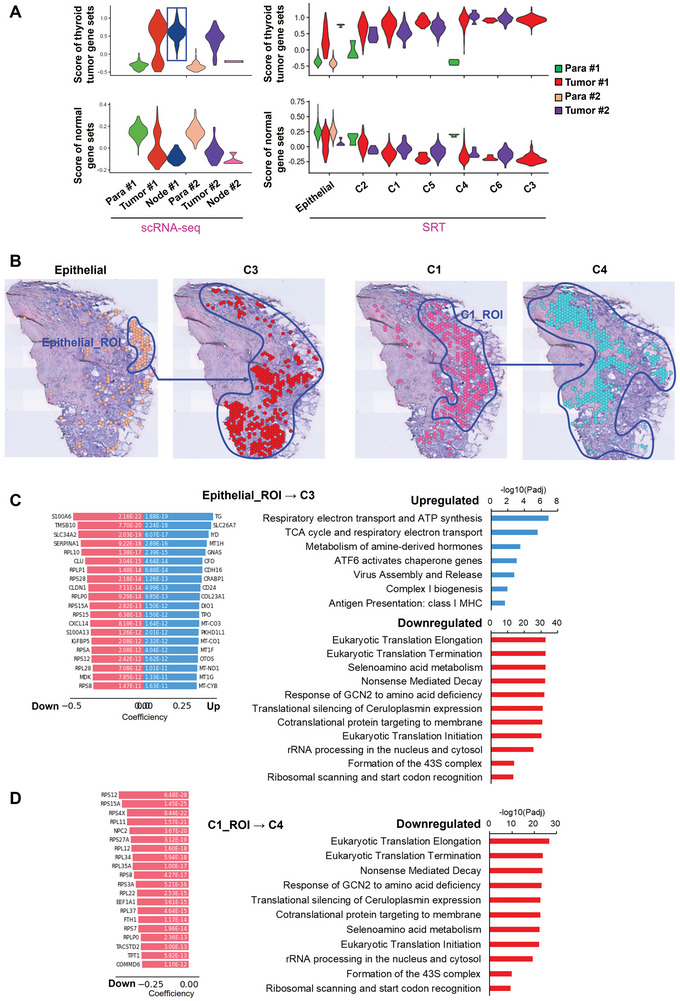
Spatial evolutionary routes and driving events in PTCs. A) Scoring of thyroid cancer and normal tissue gene sets on the annotated epithelial and PTC cluster in scRNA‐seq and all the classified spot clusters in SRT analysis. Gene sets were derived from the THCA dataset of TCGA. B) Spatial trajectory inference analysis for Tumor #1 tissue to identify evolutionary routes. Spots boxed with blue line indicate the source region for the spot cluster at the next stage. ROI, region of interest which is involved in the spatial evolutionary route. C,D) The significantly up‐ and downregulated evolution‐driving genes and enriched signaling pathways for epithelial_ROI→C3 C) route and C1_ROI→C4 route D).

The annotation for the PTC cells of scRNA‐seq and PTC spots of SRT were validated by their higher scores of thyroid tumor gene sets and lower scores of normal gene sets compared to the corresponding epithelial clusters (**Figure** **2**A and Table S2, Supporting Information). Although comparable scores were observed among the PTC spots in the primary tissues, C3 from Tumor #1 and C6 from Tumor #2 tissue had higher scores of thyroid tumor gene sets compared to other counterparts of PTC spots (Figure 2A). PTC cells in Node #1 tissue possessed the highest score of thyroid tumor gene sets compared to that in other tissues (Figure 2A).

Spatial trajectory inference analysis was then performed on all the PTC spot clusters to construct the spatial evolutionary routes of PTC cells and elaborate the driving mechanisms in Tumor #1 tissue. As a result, we observed two spatial evolutionary routes, in which a group of epithelial cells located at the edge of the tumor tissue evolved into C3 cluster, and most of the PTC cells from C1 cluster evolved into C4 cluster (Figure 2B). On the evolutionary routes, not all spots would evolve to the next stage. Thus, the regions of PTC spots involved in the spatial evolutionary routes were selected and denoted as region of interest (ROI) to identify the driving genes and signaling pathways for each evolution route (Figure 2B). During epithelial_ROI→C3, tricarboxylic acid (TCA) cycle, respiratory electron transport, complex I biogenesis, adenosine triphosphate (ATP) synthesis and class I MHC‐mediated antigen presentation were significantly upregulated, whereas mRNA translation, protein synthesis and nonsense‐mediated RNA decay were significantly downregulated (Figure 2C). Furthermore, suppressed mRNA translation may be mediated by GCN2 responding to amino acid deficiency (Figure 2C). Interestingly, all the driving genes were the downregulated genes during C1_ROI→C4 (Figure 2D). And the correspondingly downregulated signaling pathways during C1_ROI→C4 were consistent with those downregulated during epithelial_ROI→C3 (Figure 2D).

Moreover, the spots involved in the spatial evolutionary route in Tumor #1 are subjected to the analysis of cell‐cell interactions including secreting signaling, extracellular matrix (ECM)‐receptor, cell‐cell contact, heterodimers and others (Figure S3A, Supporting Information). C3 and C4 were the major spot clusters receiving signals from other spots, while fibroblast cluster was the major one sending signals out; Macrophages were also active in cell‐cell interaction (Figure S3B,C, Supporting Information). C1, C3 and C4 shared a common signal receiving pattern, which was different from that of Epithelial cluster (Figure S3D, Supporting Information). Furthermore, fibronectin 1 (FN1)‐integrin subunit alpha 3 (ITGA3), FN1‐ITGB1, ephrin‐A1(EFNA1)‐ephrin type‐A receptor 4 (EPHA4), adhesion G protein‐coupled receptor E5 (ADGRE5)‐CD55 predominantly mediated the cell‐cell interactions (Figure S3E, Supporting Information). The results of cell–cell interaction are similar to the findings recently reported by Yan et al.^[^ ^12^ ^]^

These findings indicated multiple evolutionary routes for PTC cells, including an evolution route of epithelial cells to PTC cells and another route among PTC cells. While aerobic metabolism might promote evolution toward PTC tumorigenesis, inhibition of mRNA translation and protein synthesis as well as cell‐cell interaction may be necessary for both tumorigenesis and evolution among PTC cells.

### 2.3 Different Gene Footprints Correlated with the Malignancy and Metastasis of PTC Cells

**Figure 3:**
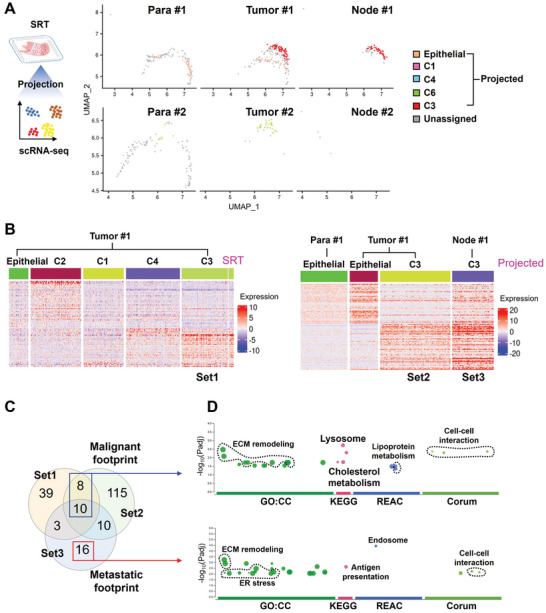
Gene footprints corelated with the malignancy and metastasis of PTC cells. A) Projection of the classified spot clusters from SRT analysis on the epithelial & PTC cell cluster from scRNA‐seq. B) Gene footprints for the original C3 spot clusters in Tumor #1 tissue from SRT analysis (Set1), the projected C3 in Tumor #1 tissue (Set2) and Node #1 tissue (Set3) from scRNA‐seq. C,D) Curated malignant and metastatic footprints derived from the overlap of Set1, Set2 and Set3 signatures C), and functional enrichment analysis for these two footprints D).

To trace the source for the metastatic PTC cells in lymph node from Patient#1, we only selected epithelial and PTC cells from scRNA‐seq, which were used as the reference cells to reversely project SRT‐identified epithelial and PTC spots of Patient#1 (**Figure** **3**A). PTC cells in Tumor #1 and Node #1 tissues were largely projected as C3 spots (Figure 3A). However, the significantly differentially expressed genes (DEGs) (Log_2_FC > 0.5, adjusted *P* < 0.05, expressed cells% > 75%) were different among the original C3 spots in Tumor #1 tissue from SRT analysis (Set1), the projected C3 spots in Tumor #1 tissue (Set2) and in Node #1 tissue (Set3) from scRNA‐seq (Figure 3B). Limited overlapped genes were observed among these three sets of gene footprints (Figure 3C).

Thus, the 18 overlapped genes between Set1 and Set2 were selected and denoted as a malignant footprint, while the 16 non‐overlapped genes of Set3 with Set1 and Set2 were denoted a metastatic footprint (Figure 3C and Table S3, Supporting Information). Functional analysis revealed that both malignant and metastatic footprints were mainly involved in ECMremodeling, organelle functions and cell‐cell interactions (Figure 3D). Different from malignant footprint, metastatic footprint was also involved in endoplasmic reticulum stress and antigen presentation (Figure 3D). The lipid metabolism enriched in the malignant footprint was not found in the metastatic footprint (Figure 3D). Lipid metabolism disorder plays an important role in the tumor process, and ferroptosis caused by lipid peroxidation is one of the barriers that cancer cell evolution, especially the acquisition of drug resistance, needs to overcome.^[^ ^13^ ^]^ Therefore, this observation may suggest that ferroptosis is suppressed with the metastatic evolution of PTC cells.

**Figure 4:**
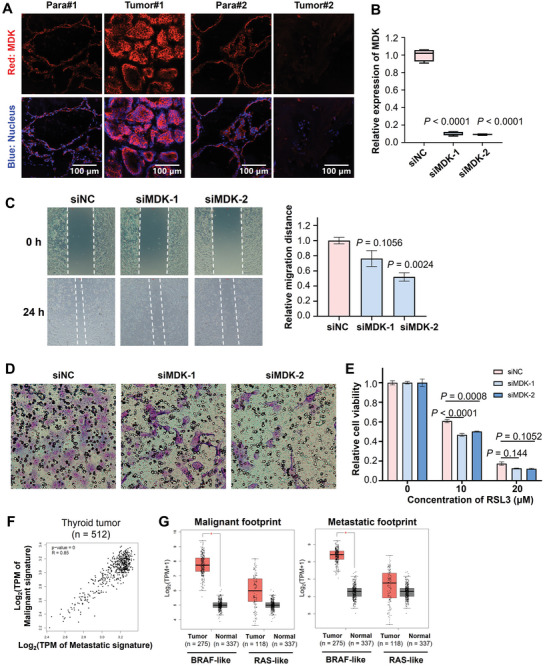
Validation of curated malignant and metastatic footprints of PTCs. A) Immunofluorescent assay of MDK from metastatic footprint in carcinoma tissues and matched para‐carcinoma tissues. B) The knockdown efficiency of the MDK gene in B‐CPAP cells as detected by RT‐qPCR assay. B‐CPAP cells were transfected with two siRNAs targeting MDK (siMDK‐1 and ‐2) and the negative control (siNC). C) The migration ability of B‐CPAP cells as measured by a wound healing assay. The relative migration distances for each group are presented in bar graph on the right panel. D) The invasion ability of B‐CPAP cells as determined using a Matrigel‐coated Transwell assay. Cells that penetrated the matrigel were stained with Wright‐Giemsa. E) The alteration of cell viability of B‐CPAP cells upon treatment of RSL3, a classical ferroptosis inducer, with or without knockdown of the MDK gene. F) Correlation analysis between malignant and metastatic footprints in thyroid tumor patients from THCA dataset. G) The validation of the malignant and metastatic footprints by the THCA dataset.

Immunofluorescence assay demonstrated that the protein content of Midkine (MDK), one of the top DEGs from these two footprints, increased in Tumor#1 compared to Para#1; however, MDK protein content decreased in Tumor#2 compared to Para#2 (**Figure** **4**A). MDK was reported to promote metastasis by its mitogenic, pro‐inflammatory and angiogenic functions in several tumor types including melanoma, head, and neck cancers.^[^ ^14^ ^]^ We next validated the effect of MDK on the oncology and ferroptosis of PTCs using the B‐CPAP cells (a cell line with a BRAF^V600E^ mutation^[^ ^15^ ^]^). Firstly, we used small interfering RNA (siRNA) to knockdown the expression of MDK (Figure 4B). Wound healing assay (Figure 4C) and Trans‐well assay (Figure 4D) demonstrated that knockdown of MDK expression significantly impaired the migration and invasion abilities of B‐CPAP cells. Moreover, MDK knockdown significantly enhanced the decrease of cell viability induced by RSL3, a classical ferroptosis inducer (Figure 4E). These results indicates that MDK may have the potential to enhance the growth and metastasis of PTC cells, in which increasing ferroptosis resistance of PTC cells maybe one of the underlying mechanisms. This is consistent with the finding that Patient 1#, who had high MDK expression in tumor tissue and exhibited lymph node metastasis.

The clinical value of malignant and metastatic footprints was also tested using the thyroid carcinoma (THCA) dataset from the Cancer Genome Atlas (TCGA). A significant positive correlation was observed between malignant and metastatic in thyroid cancer patients (Figure 4F). Both the malignant and metastatic footprints were significantly increased in BRAF‐like thyroid cancer samples compared to the normal samples (Figure 4G). The widespread clinical predictive values of the malignant and metastatic footprints were also validated in multiple cancer datasets from TCGA, such as pancreatic adenocarcinoma, thymoma, glioblastoma multiform, renal clear cell carcinoma and hepatocellular carcinoma (Figure S4, Supporting Information).

### 2.4 Ferroptosis Resistance Contributed to PTC Evolution

**Figure 5:**
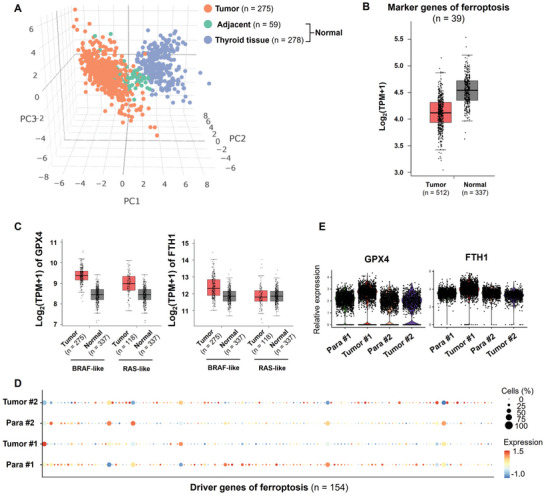
Ferroptosis resistance of PTC cells. A,B) PCA dimension reduction A) and the expression levels B) of 39 ferroptosis marker genes in normal, para‐carcinoma and thyroid cancer tissues from the THCA dataset of TCGA database. C) The expression levels of GPX4 and FTH1 in thyroid cancer and normal tissue samples from the THCA dataset. D,E) The expression levels of 154 ferroptosis driver genes D) and GPX4 and FTH1 E) in the para‐carcinoma and thyroid cancer tissues from Patient#1 and Patient#2.

As discussed above, ferroptosis inhibition may be involved as suggested by differentially enrichment of lipid metabolism between the malignant and metastatic footprints (Figure 3D); and we also found that RSL3 can reduce the viability of PTC cells (Figure 4E). Therefore, we curated 39 ferroptosis marker genes from the public database FerrDb,^[^ ^16^ ^]^ and firstly analyzed the expression of these 39 ferroptosis marker genes in the THCA dataset from the TCGA database. As a result, the expression levels of these 39 genes could well distinguish between normal thyroid tissue, peritumoral tissue, and tumor tissue (**Figure** **5**A), indicating that ferroptosis is one of the key events in the development of thyroid cancer. Further analysis found that the expression levels of these ferroptosis marker genes were significantly lower in thyroid tissues than in normal tissues (Figure 5B). Previous studies have shown that high expression of Glutathione peroxidase 4 (GPX4) and ferritin heavychain 1 (FTH1) is an important prerequisite for 27‐Hydroxycholesterol‐driven ferroptosis resistance and subsequently drug resistance acquirement in breast cancer.^[^ ^13^ ^]^ The analysis on the THCA dataset also revealed that, compared to normal tissue, the expression level of GPX4 was significantly upregulated in both BRAF and RAS thyroid cancer tissues, while the expression of FTH1 was also significantly upregulated in BRAF thyroid cancer tissues (Figure 5C).

Importantly, we observed the suppressed expression of the 39 ferroptosis marker genes in Tumor#1 compared to Para#1 (Figure 5D). However, the expression of these ferroptosis marker genes was mildly upregulated in Tumor#2, at least comparable, compared to Para#2 (Figure 5D). Consistently, an increase in the expression levels of GPX4 and FTH1 observed in in Tumor#1 compared to Para#1 but not in Tumor#2 compared to Para#2 (Figure 5E). These results revealed that ferroptosis resistance may contributed the evolution of PTC cells.

## 3 Discussion

Recently, Saiselet and colleagues performed the SRT analysis on a tumor sample from a PTC patient using the first‐generation spatial transcriptomics, and revealed spatial variation of several classic PTC marker genes, cell types and cell states across the PTC tissue,^[^ ^11^ ^]^ demonstrating the power of SRT analysis in delineating the morphology‐associated gene expression footprints in PTCs. However, Saiselet et al. did not demonstrate enough spatial information and PTC evolution,^[^ ^11^ ^]^ and the 1st generation of SRT technique had a resolution of 100 µm over ≈1000 spots.^[^ ^17^ ^]^ In the current study, we used an upgraded SRT technique, offering a resolution of 55 µm over ≈5000 spots, and comprehensively revealed the spatial heterogeneity and features and spatial evolutionary routes of PTCs.

Accumulating evidence suggests that besides glycolysis, mitochondrial metabolism plays as a central metabolic organelle required for tumorigenesis by generating ATP and restoring TCA cycle intermediates for macromolecule synthesis in cancer cells.^[^ ^18^ ^]^ Monocarboxylate transporter 1 and 4 for lactate, and the transporter of the outer mitochondrial membrane TOMM20 were revealed to be associated with the reverse Warburg effect in the cancer cells and stromal cells from non‐malignant thyroid tissue, PTC tissue and anaplastic thyroid cancer (ATC) tissue.^[^ ^19^ ^]^ A reverse Warburg effect in PTCs was supported by the increased mitochondrial oxidative metabolism in PTC cells.^[^ ^20^ ^]^ Our results in the driving events for the spatial evolution of PTC cells demonstrated the upregulation of mitochondrial functions such as the citric acid cycle, electron transport, and ATP synthesis, which would further verify the reverse Warburg effect in PTCs and more importantly demonstrated its contribution in PTC intertumoral evolution. Both aberrant translation and post‐translational protein modifications are also important in the pathogenesis of various cancers, and thus targeting mRNA translation has become a potential strategy for cancer treatment.^[^ ^21^ ^]^ General control non‐derepressible 2 (GCN2)‐activating transcription factor 4 pathway is critical for tumor cell survival and proliferation upon nutrient deficiency.^[^ ^22^ ^]^ Moreover, irregulated nonsense‐mediated RNA decay, both hyper‐activation and hyper‐inhibition, are implicated in cancer progression.^[^ ^23^ ^]^ In the current study, analysis of the driving genes and signaling pathways involved in the evolutionary paths revealed the downregulation of protein synthesis processes, which may be mediated by GCN2 responding to amino acid deficiency.

In recent years, a number of risk genes and gene sets of PTCs have been identified and constructed based on clinical and experimental studies as well as bioinformatic analysis based on bulk RNA‐seq.^[^ ^8^, ^10^, ^24^ ^]^ Herein, we curated two spatial‐related gene footprints which may help predict the malignancy and metastasis of PTC cells. There was no significant difference in the enriched molecular events and signalling pathways between these two gene fingerprints, mainly involving functions such as ECM remodelling, cell interactions, lysosomes, and endocytosis. However, the signal pathways related to lipoprotein and cholesterol metabolism, which were indicated in the malignant footprint, were not present in the metastatic footprint. Following this clue, we validated the issue of ferroptosis resistance in PTC cells. Ferroptosis is distinct from other programmed cell deaths such as apoptosis, necrosis, and autophagy.^[^ ^25^ ^]^ In recent years, increasing evidence has shown that ferroptosis is extensively involved in various pathological processes and the development of diseases.^[^ ^26^ ^]^ Resistance to ferroptosis is one of the important mechanisms underlying the resistance to chemotherapy, radiotherapy, and immunotherapy in cancer treatment.^[^ ^27^ ^]^ The expression characteristics of ferroptosis‐related genes have been reported to serve as independent prognostic indicators for thyroid cancer prognosis.^[^ ^28^ ^]^ Additionally, high expression of GPX4 and FTH1 are crucial prerequisites for ferroptosis resistance in breast cancer cells in acquiring drug resistance.^[^ ^13^ ^]^

Our study primarily investigated the spatial evolution of PTC cells, detailing the mechanisms driving malignancy and metastasis, with the emphasis on the evolutionary routes of PTC cells and their metabolic adaptations as discussed above. Yan et al. centered on prognosis‐associated cellular heterogeneity within the PTC microenvironment, specifically identifying how different cell types contribute to tumor behavior and patient outcomes.^[^ ^12^ ^]^ Specific ligand‐receptor interactions and cellular transitions (e.g., atypical follicular cells to tumor cells) that correlate with prognosis were identified, with emphasis on the role of cellular interactions in shaping the tumor microenvironment and influencing patient survival.^[^ ^12^ ^]^ Our study also indicated the involvement of cell‐cell interactions in the spatial evolution of PTC cells.

Another study developed a diagnostic model based on metastasis‐associated cancer‐associated fibroblasts (CAF) genes that can predict LNM with high accuracy.^[^ ^29^ ^]^ In particularly, CD36 expression in CAFs was demonstrated to promote PTC cell proliferation, migration, invasion and inhibits apoptosis.^[^ ^29^ ^]^ CAFs were also identified in the current study, and we observe the potential contribution of CAFs to the spatial evolution of PTCs via cell‐cell interaction. However, the two gene footprints correlated with PTC malignancy and metastasis were not specific to CAFs. The study from Pu et al. focused on scRNA‐seq data from a larger cohort of patients (11 patients), analyzing the entire clinical course of PTC, including para‐tumors and metastatic sites, while our study integrated SRT with scRNA‐seq to provide spatial context to the cellular heterogeneity observed.^[^ ^30^ ^]^ Pu et al. categorized thyrocytes into distinct phenotypes (follicular‐like, partial epithelial–mesenchymal transition‐like, and dedifferentiation‐like) and discusses their implications for tumor characteristics and responses to treatment, particularly in relation to RAI therapy.^[^ ^30^ ^]^

Wang et al. revealed that different tumor sites within the same patient can exhibit distinct cellular compositions and signaling pathways, even when sharing genetic mutations like BRAF^V600E^.^[^ ^31^ ^]^ They further elaborated on the dynamics of T cells and macrophages, particularly the increase of M2‐like macrophages in advanced disease stages,^[^ ^31^ ^]^ which aligns with the our's findings on spatial heterogeneity and high infiltration of immune cells. Wang et al. also investigated inter‐tumor heterogeneity across multiple tumor samples from different lobes of the thyroid, providing a broader perspective on how bilateral PTC can differ at a transcriptomic level, but without insight on the spatial transcriptomics.^[^ ^31^ ^]^ On the other hand, we curated two gene footprints associated with malignancy and metastasis, focusing on their functional implications in PTC evolution. Wang et al. constructed a specific 6‐gene signature related to the cytokine‐cytokine receptor interaction pathway, which serves as a prognostic indicator for patient survival, showcasing a more clinical application of their findings.^[^ ^31^ ^]^

## 4 Conclusion

To summarize, we combined scRNA‐seq and SRT to explore the unrecognized mechanisms underlying the evolution of PTCs. Our results demonstrated greater spatial heterogeneity and multiple spatial evolution routes of PTC cells in the primary PTC tissue with lymph node metastasis. Two gene footprints were curated to be closely correlated with the malignancy and metastasis of PTCs. Ferroptosis resistance may contribute to PTC evolution.

## 5 Experimental Section

### Patients and Sample Collection

Papillary thyroid carcinoma tissues, matched para‐carcinoma tissues and lymph node tissues were collected from one PTC patient with lymph node metastasis and another PTC patient without metastasis at the Department of General Surgery, Peking Union Medical College Hospital. Patients provided informed consent. Patients were enrolled at their initial visit, and samples were collected before surgery and treatment (Baseline characteristics of the patients and tumor samples are shown in Table S1, Supporting Information). Inclusion criteria: preoperative and intraoperative diagnosis of thyroid cancer with histopathological subtype being papillary carcinoma; with or without cervical lymph node metastasis. Exclusion criteria: distant metastases; cases after surgery or other treatments, cases of recurrent PTC. All procedures were approved by the ethics committee at Peking Union Medical College Hospital with ethical approval number I‐22PJ232. All diagnoses were verified by histological review by Junior Pathologists.

All tissues were freshly collected from patients during surgical operation prior to treatments, and temporarily stored in ice‐cold serum‐free RPMI‐1640 medium (Corning). For SRT, part of the tissue for each specimen was cut into appropriate sizes, washed in ice‐cold DPBS, embedded in optical cutting tissue (OCT) (Tissue‐Tek, Sakura Finetek USA Inc., Torrance, CA) and flash‐frozen in an isobutane bath surrounded in liquid nitrogen. The rest of tissue from each specimen was washed in ice‐cold PBS to remove blood, and then minced with a scalpel to pieces ≈3 mm diameter in ice‐cold cell‐culture dish. For each specimen, 4–5 pieces were stored in a cell cryopreservation tube with 1.5 mL Tissue Storage Solution (MACS, Bergisch Gladbach, Germany) at the temperature of 2–8 °C, followed by scRNA‐seq library construction within 48 h.

### Library Preparation, Sequencing and Data Preprocessing for scRNA‐Seq Data

All six samples were immediately subjected to tissue preparation and single cell dissociation followed by droplet‐based scRNA‐seq using the 10X Genomics Chromium Single Cell 3′ platform v3 according to manufacturer's instructions. The library was sequenced on BGISeq‐500 with paired‐end sequencing. Raw reads were aligned to the hg38 human transcriptome (UCSC) and preprocessed using the CellRanger v3.0.2 pipeline (10x Genomics) with default parameters.

### Bioinformatic Analyses for scRNA‐Seq Data

The gene‐barcode count matrices generated by CellRanger were used as input for downstream analyses by Seurat package (version 4.0.3).^[^ ^34^ ^]^ In details, for the quality control, low expression genes expressed in less than 3 cells was excluded. Cells with more than 200 genes and less than 5000 genes and less than 10% of reads mapped to mitochondrial genes were retained. Scores of cell cycle and percentages of mitochondrial and ribosomal genes were regressed out to remove the bias impact of cell cycle, mitochondria and ribosomes on the downstream analyses. The SC Transform(), a new modeling function introduced by Seurat for normalization that effectively removes technical variation while preserving biological heterogeneity, was used to normalize and scale the data to a common level, often assuming a set number of UMIs per cell. DoubletFinder package was implemented to evaluate the impact of doublets, which were artifacts resulting from the fusion of two cells during the sequencing process, on the downstream analyses.^[^ ^35^ ^]^ This package employs various computational strategies, such as the analysis of gene expression patterns and cellular markers, to predict and address the doublet formation.

Dimensional reduction techniques including PCA (Principal Component Analysis), t‐SNE (t‐Distributed Stochastic Neighbor Embedding), and UMAP (Uniform Manifold Approximation and Projection) were used to visualize high‐dimensional data in two or three dimensions via functions RunPCA(), RunTSNE(), and RunUMAP() from Seurat, respectively. Highly variable features were used for PCA. UMAP plots were obtained by reducing 30 principal components at a resolution of 1.2. The FindClusters() function was used to partition the data into clusters based on the results of dimensional reduction. To find markers that are differentially expressed between clusters, the FindMarkers() function from Seurat, which by default performs a non‐parametric Wilcoxon rank sum test was used to determine the classification power of individual markers. We employed several functions from Seurat to visualize the data, including DimPlot() to visualize reduced dimension, VlnPlot() for violin plots of gene expression across clusters, FeaturePlot() to visualize feature expression in low‐dimensional space, and DoHeatmap() for heatmaps of gene expression.

The Integration and Label Transfer strategy from Seurat to amalgamate our scRNA‐seq datasets derived from the 6 samples was utilized. This strategy was particularly adept at harmonizing the data from different sources, accounting for technical and biological variability, and preserving the underlying structure of the individual datasets. The Label Transfer feature can project the annotations from one dataset onto the integrated dataset, enabling the identification of conserved and distinct cellular populations.

### Library Preparation, Sequencing and Data Preprocessing for SRT Data

Two frozen sections (10 µm) per tissue were mounted onto 10x Genomics Visium slide and processed according to manufacturer's instructions. After fixation with methanol, the tissues were stained with hematoxylin and eosin and imaged with Bright‐field imaging, followed by tissue permeabilization for 10 min. Reverse transcription was performed on the slides to synthesize the first strand cDNA, which was then quantified and subjected to generate the libraries according to the standard workflow provided the manufacturer's protocol.

Spatial alignment of tissue H&E image and barcode location were created by Loupe (10x Genomics) with manual selection of the tissue overlayed region. The aligned tissue region barcode and sequence files were then processed and aligned to hg38 human transcriptome (UCSC) using the SpaceRanger v1.2.1 pipeline (10x Genomics).

### Bioinformatic Analyses for SRT Data

No prefiltering was performed on the genes and spots after data preprocessing by SpaceRanger pipeline. The gene‐spot count matrices generated by SpaceRanger were subjected to the Seurat package for downstream analyses. Regressing out of cell cycle, mitochondrial and ribosomal genes, data normalize and scale, PCA, UMAP dimensionality reduction and visualization were performed on SRT data as described in scRNA‐seq analysis. Four SRT datasets from all samples were integrated using the Merge() function from Seurat package. This function allows for the preservation of both raw and normalized data matrices, ensuring that the integrated dataset is representative of the underlying biology. Following the integration, FindTransferAnchors() function in Seurat was used to perform the projection of cell clusters between scRNA‐seq and SRT with ‘score.filter = 0.55′, ensuring that only the most confidently matched cell clusters were transferred. The label transfer process is facilitated by the TransferData() function, which aligns the query dataset to the reference dataset based on the identified anchors. This alignment allows for the inference of cell identities in the query dataset based on the known annotations of the reference dataset, thus enabling a comparative analysis across different types of datasets.

### Cell Annotation

In order to annotate cell clusters, it was collected gene markers for immune cells, stromal cells, epithelial cells and thyroid cancer cells from several datasets, including PanglaoDB (https://panglaodb.se/), LM22 of CIBERSORT (https://cibersort.stanford.edu/), GEPIA2 (http://gepia2.cancer‐pku.cn/) and THCA of TCGA (https://portal.gdc.cancer.gov/). AddModuleScore() function in Seurat package was implemented to calculate the average expression of gene markers on all classified cell clusters from scRNA‐seq and SRT data. Cell clusters were then annotated as the cell type with the highest score.

### Differential Expression Analysis and Functional Enrichment Analysis

Differential expression analysis was performed by the FindMarkers() or FindAllMarkers() function in Seurat with ‘wilcox’ method. Assay “RNA” for scRNA‐seq or “Spatial” for SRT was used in differential expression analysis on the corresponding normalized data. DEGs with Log_2_FC > 0.5, adjusted Bonferroni corrected *P* < 0.05 and expressed cells% > 75% were selected to construct the gene footprints. Functional enrichment analysis was performed using an online tool, g:GOSt of g:Profiler.^[^ ^36^ ^]^

### Spatial Trajectory Inference Analysis

stLearn package was implemented to perform spatial trajectory inference analysis on SRT data with the default parameters.^[^ ^37^ ^]^ Spots from the Epithelial cluster were strategically chosen as root spots, providing a foundational reference point for the analysis. The pseudo‐time‐space (PSTS) method from stLearn was run on global level to construct the spatial trajectories among cell clusters. This method was designed to infer the order of transcriptomes along a trajectory, allowing for the visualization of dynamic processes such as cell differentiation, activation, or even disease progression across tissue space and time. The PSTS algorithm calculates pseudo‐temporal values for each spot, integrating both gene expression and spatial information to provide a nuanced view of cellular dynamics within the tissue. The highly correlated genes with the PSTS values were collected as driving genes based on the spatial trajectories and tree plot. These genes were identified based on their significant correlation with the PSTS values, as determined through statistical analysis such as Spearman's rank correlation test.

### Cell–Cell Interaction Analysis

CellChat was implemented to perform cell‐cell interaction analysis on SRT data and visualization with the default parameters.^[^ ^38^ ^]^ This package employs a mass‐action‐based model to quantify the communication probability between cell clusters, considering ligand‐receptor interactions, including those with multi‐subunit structures, and their modulation by cofactors. We utilized its database, CellChatDB, which is manually curated and includes interactions supported by literature, covering a wide range of signaling pathways such as secreting signaling, ECM‐receptor interactions, cell‐cell contact, heterodimers, KEGG pathways, and literature‐curated interactions.

### Immunofluorescent Assay

The OCT‐embedded frozen tissue was sectioned into 10‐µm‐thick slices and mounted onto glass slides. The sections were then fixed in 4% paraformaldehyde (Servicebio, China) for 30 min. After fixation, the sections were rinsed in PBS with gentle shaking three times for 5 min each. Permeabilization was performed using PBS containing 0.5% Triton X‐100 (Solarbio, China) for 10 min, followed by washing the sections three times in PBST for 5 min each. Subsequently, the sections were blocked with bovine serum albumin (BSA, Biosharp, China) for 30 min. Anti‐MDK (diluted 1:50, ABclonal, China) antibodies was applied to the sections, and incubation was carried out overnight at 4 °C. After incubation, the sections were washed three times with PBS, each wash lasting 5 min.

Next, Alexa Fluor 594‐conjugated goat anti‐rabbit IgG secondary antibody (diluted 1:500, Abcam, UK) was added, and the sections were incubated for 30 min at 30 °C in the dark. All subsequent steps were performed in the dark. The sections were washed three times with PBS for 3 min each and then stained with DAPI (ZKWB‐Bio, China) for 10 min to label the nuclei. After staining, the sections were washed three more times with PBS and mounted with a mounting medium (ZKWB‐Bio, China). The labeled sections were imaged using an FV12‐IXCOV confocal microscope (Olympus, Japan) and images were processed using a cellSens software (Olympus, Japan).

### siRNA Transfection, Wound Healing and Transwell Assay, and Ferroptosis Assay

B‐CPAP thyroid cancer cells were obtained from Procell Life Science & Technology Co., Ltd. (Wuhan, China) and cultured in RPMI 1640 medium supplemented with 10% fetal bovine serum (FBS) and 1% penicillin‐streptomycin at 37 °C in a humidified atmosphere containing 5% CO₂. To modulate MDK gene expression, small interfering RNA (siRNA) targeting MDK and the corresponding negative control sequences were synthesized by HIPPOBIO (China). According to the manufacturer's protocol, the dissolved siRNA was gently mixed with Lipofectamine 2000 (Invitrogen, USA), which had been diluted in serum‐free RPMI 1640 medium, and incubated at room temperature for 20 min. The siRNA‐Lipofectamine 2000 complexes were then added to the cells. After incubating for 5 h at 37 °C, the medium was replaced with RPMI 1640 containing 10% FBS and 1% penicillin‐streptomycin, and the cells were cultured for an additional 24 h.

Following transfection, cells were trypsinized and seeded into 24‐well plates for Wound healing assay, into the upper chamber of Transwell inserts for invasion assay, and into 96‐well plates for cell viability assay. For the Wound healing assay to evaluate cell migration, cells were seeded at a density of 90%. A straight scratch was introduced into the monolayer using a pipette tip, following a ruler for guidance. The culture medium was then replaced with serum‐free RPMI 1640, and images of the scratch were captured under a microscope (Carl Zeiss, Germany). After 24 h, the scratch was imaged again, and the migration distance was calculated as the difference between the initial and final scratch widths. Data were normalized to the negative control group.

For the Transwell assay to assess cell invasion ability, Matrigel (Corning, USA) was diluted in serum‐free RPMI 1640 medium at a ratio of 1:8, and 60 µL of the diluted Matrigel was added to the upper chamber of 24‐well Transwell inserts. The inserts were incubated at 37 °C for 3 h to allow the Matrigel to solidify. After removing excess liquid, 100 µL of serum‐free RPMI 1640 was added into the upper chamber to hydrate the Matrigel. Cells were seeded into the upper chamber at a density of 5 × 10^4^ cells per well in serum‐free RPMI 1640, while 650 µL of RPMI 1640 containing 10% FBS was added to the lower chamber. After 48 h, non‐invading cells on the upper surface of the membrane were removed using a cotton swab. Cells that had invaded through the Matrigel were stained with Giemsa‐Romanowsky and visualized under a microscope (Carl Zeiss, Germany).

For the cell viability assay to investigate the role of MDK in ferroptosis resistance in B‐CPAP cells, cells were seeded into 96‐well plates at 70% confluence. After overnight incubation to allow adherence, cells were treated with varying concentrations of RSL3 (0–20 µM; MCE, China) diluted in RPMI 1640 containing 10% FBS. After 24 h, Resazurin (MCE, China) was added to assess cell viability using the Alamar Blue assay. The fluorescence in the cell supernatant was measured using a microplate reader at an excitation/emission wavelength of 530/590 nm, and the data were normalized to values from cells not treated with RSL3 in each transfection group, respectively.

## Conflict of Interest

The authors declare no conflict of interest.

## Author Contributions

G.Z. and S.C. contributed equally to this work. G.Z. and S.C. carried out the experiments. G.Z., S.C. and S.Z. performed the bioinformatic analyses. W.M., Q.W. and L.S. contributed to the experiments. C.Z. and G.C. revised the manuscript. S.C. and S.Z. designed the study and wrote the manuscript.

## Supporting information



Supporting Information

Supporting Information

Supporting Information

## Data Availability

The data that support the findings of this study are openly available in National Genomics Data Center, China National Center for Bioinformation/Beijing Institute of Genomics, Chinese Academy of Sciences at https://ngdc.cncb.ac.cn/gsa‐human, reference number HRA003726.
