# Supplementary material for: Spatial and Single‐Cell Transcriptomics Unraveled Spatial Evolution of Papillary Thyroid Cancer
Source: Adv Sci (Weinh). 2024 Nov 14;12(2):2404491. doi: 10.1002/advs.202404491 (PMC11727256; doi:10.1002/advs.202404491)
Supplement: Supplementary file 1 — Supporting Information [file ADVS-12-2404491-s002.docx]

**Table S1. Information of patients and tumor samples.**

|  | **Gender** | **Age** | **Tumor** | | | **TNM stage** | | |
| --- | --- | --- | --- | --- | --- | --- | --- | --- |
|  |  |  | **Size** | **Type** | **BRAFV600E** | **T** | **N** | **M** |
| Patient#1 | Male | 28 | 2 lesions with 0.1 cm or 0.8 cm in diameter | Classic PTC | (+) | T1a | N1b | M0 |
| Patient#2 | Male | 32 | 0.8 cm in diameter | Classic PTC | (+) | T1a | N0 | M0 |

**Table S2. Gene lists of the thyroid cancer and normal tissue genes curated from THCA gene set of TCGA.**

| **Thyroid cancer** | **Normal tissue** | | |
| --- | --- | --- | --- |
| ANXA1 | AHSA2 | HBA2 | RMST |
| APOC1 | AKAP8L | HBB | RPS4Y1 |
| C19orf33 | AP1G2 | HOOK2 | SERPINF1 |
| CCND1 | APOD | INPPL1 | SLC26A7 |
| CD55 | ARGLU1 | LENG8 | SOD3 |
| CHI3L1 | ATHL1 | LINC00969 | SPARCL1 |
| CITED1 | C1QTNF1 | MALAT1 | STAG3L5P |
| CLDN1 | C1R | MAN2C1 | TAGLN |
| CST6 | C1S | MAT2A | TFF3 |
| CTSH | C7 | MBD6 | TNS2 |
| DUSP6 | CCL14 | MT1F | TPM2 |
| FN1 | CCL21 | MT1G | TPO |
| KRT19 | CCNL1 | MT1H | TSPYL2 |
| LGALS3 | CCNL2 | MYO15B |  |
| MDK | CFD | NDRG2 |  |
| NPC2 | CLASRP | NEAT1 |  |
| PDLIM4 | COL6A1 | NNMT |  |
| PROS1 | CORO6 | NPIPA1 |  |
| S100A1 | CRABP1 | NPIPB3 |  |
| S100A6 | DCN | NPIPB5 |  |
| SDC4 | EFEMP1 | PAN2 |  |
| SERPINA1 | EPOR | PAX8 |  |
| SFTPB | FABP4 | PILRB |  |
| SLC34A2 | FAM193B | PKHD1L1 |  |
| SLPI | FBLN1 | PLA2G6 |  |
| TACSTD2 | GOLGA8A | PLCG1 |  |
| TNFRSF12A | GOLGA8B | PLEKHH1 |  |
| XIST | GSTM2 | PNISR |  |
| ZCCHC12 | HBA1 | RAP1GAP |  |

**Table S3. Gene lists of the constructed malignant and metastatic footprints.**

| **Malignant footprint** | **Metastatic footprint** |
| --- | --- |
| AGR2 | AOPEP |
| ANXA1 | ARHGDIB |
| APOC1 | B2M |
| ARMCX3 | COL8A1 |
| CHPT1 | CTSS |
| CTSC | EEF1A1 |
| CTSH | MDK |
| ECRG4 | NRP2 |
| ENTPD1 | PCSK2 |
| IGSF1 | PDLIM1 |
| LGALS3 | PDZK1IP1 |
| NMB | PROS1 |
| NPC2 | RPS29 |
| TACSTD2 | SERPINA1 |
| TESC | SLC34A2 |
| TPD52L1 | SSR3 |
| TSC22D1 |  |
| ZCCHC12 |  |

**Supplementary Figures**


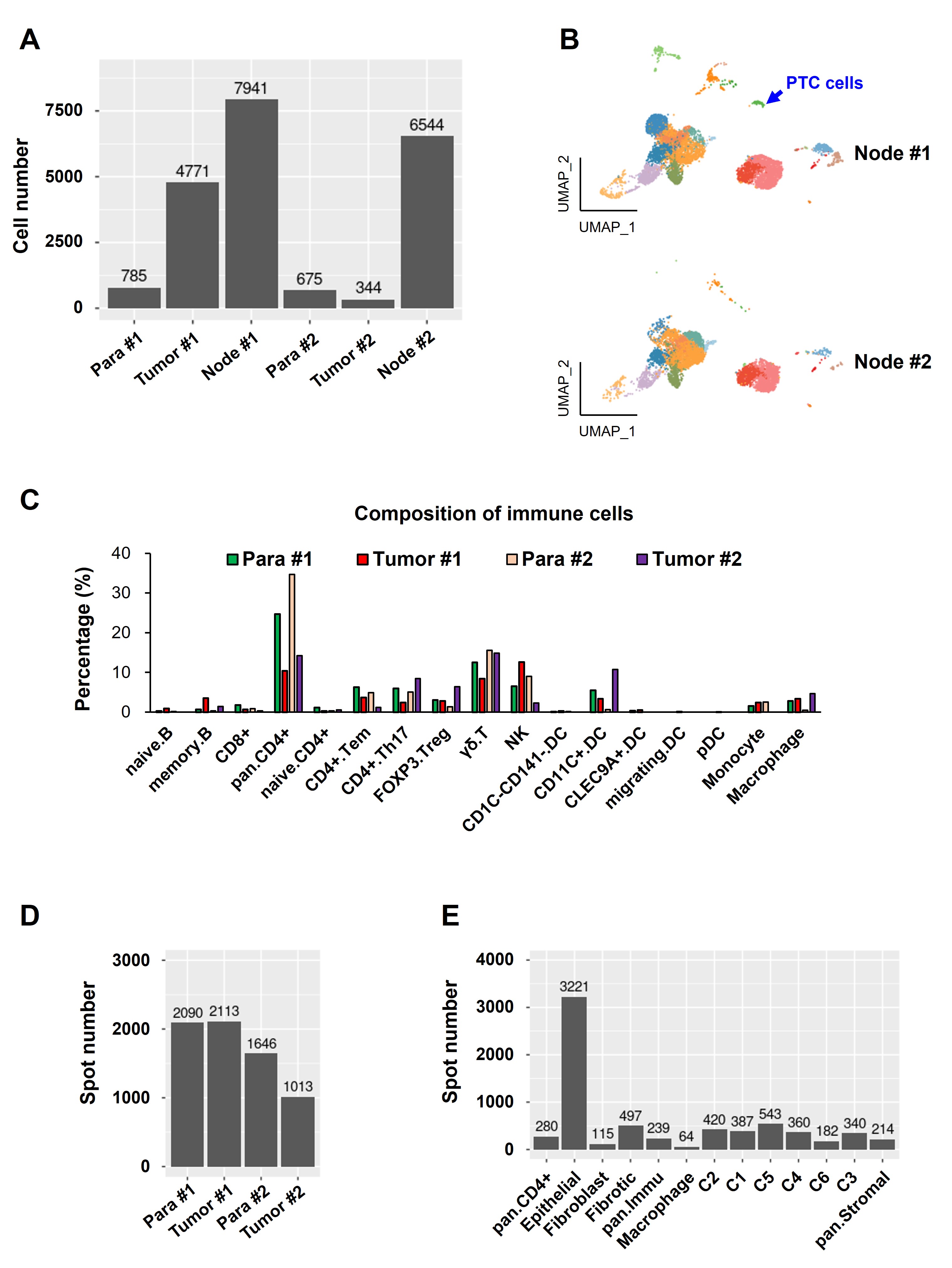


**Figure S1. (A) Cell numbers, (B) UMAP visualization and (C) immune composition of the annotated cell types in scRNA-seq. (D-E) Spot numbers of the annotated spots in SRT.** Blue arrow indicates the metastatic PTC cells in the lymph node tissue in (B).


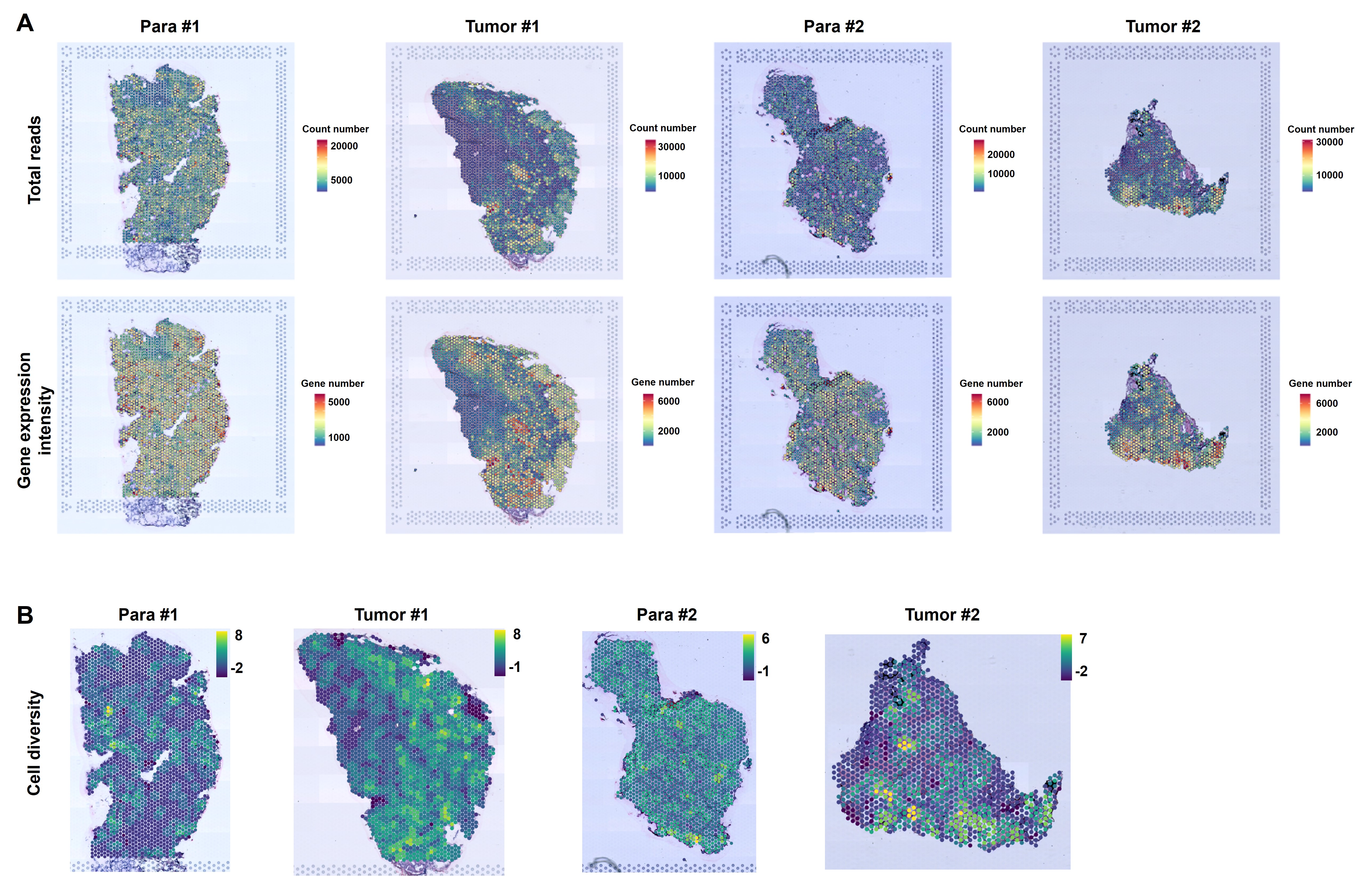


**Figure S2. Spatial visualization of cell heterogeneities as demonstrated by the number of counts and genes (A) and cell diversities (B).**


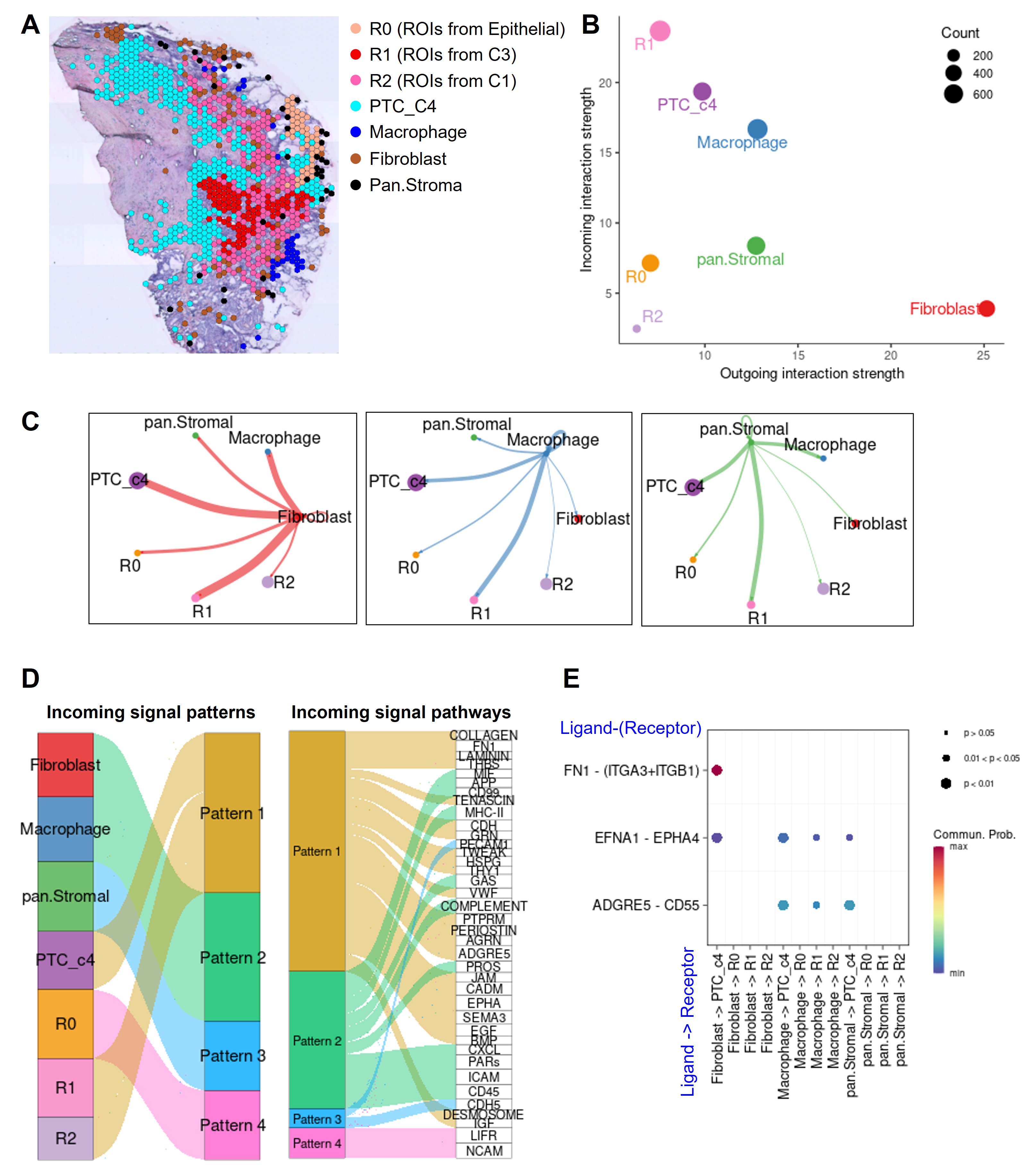


**Figure S3. Cell-cell interactions may contribute to PTC evolution.**

(A) Spots involved in the spatial evolutionary route selected for the cell-cell interaction analysis and these spots are denoted as ROIs (region of interest), including ROIs from Epithelial (labelled as R0), C3 (labelled as R1) and C1 clusters (labelled as R2) as well as all spots from C4 (labelled as PTC_C4). Cell-cell interactions include secreting signaling, ECM-receptor, cell-cell contact, heterodimers and other modes. (B-C) Visualization of signal senders (outgoing interaction strength) and receivers (incoming interaction strength). Line thickness indicates the interaction numbers in (C). (D) The cell-cell interaction patterns and the corresponding signaling pathways for the incoming signals. (E) The dominant ligand-receptor pairs mediating the cell-cell interactions.

**
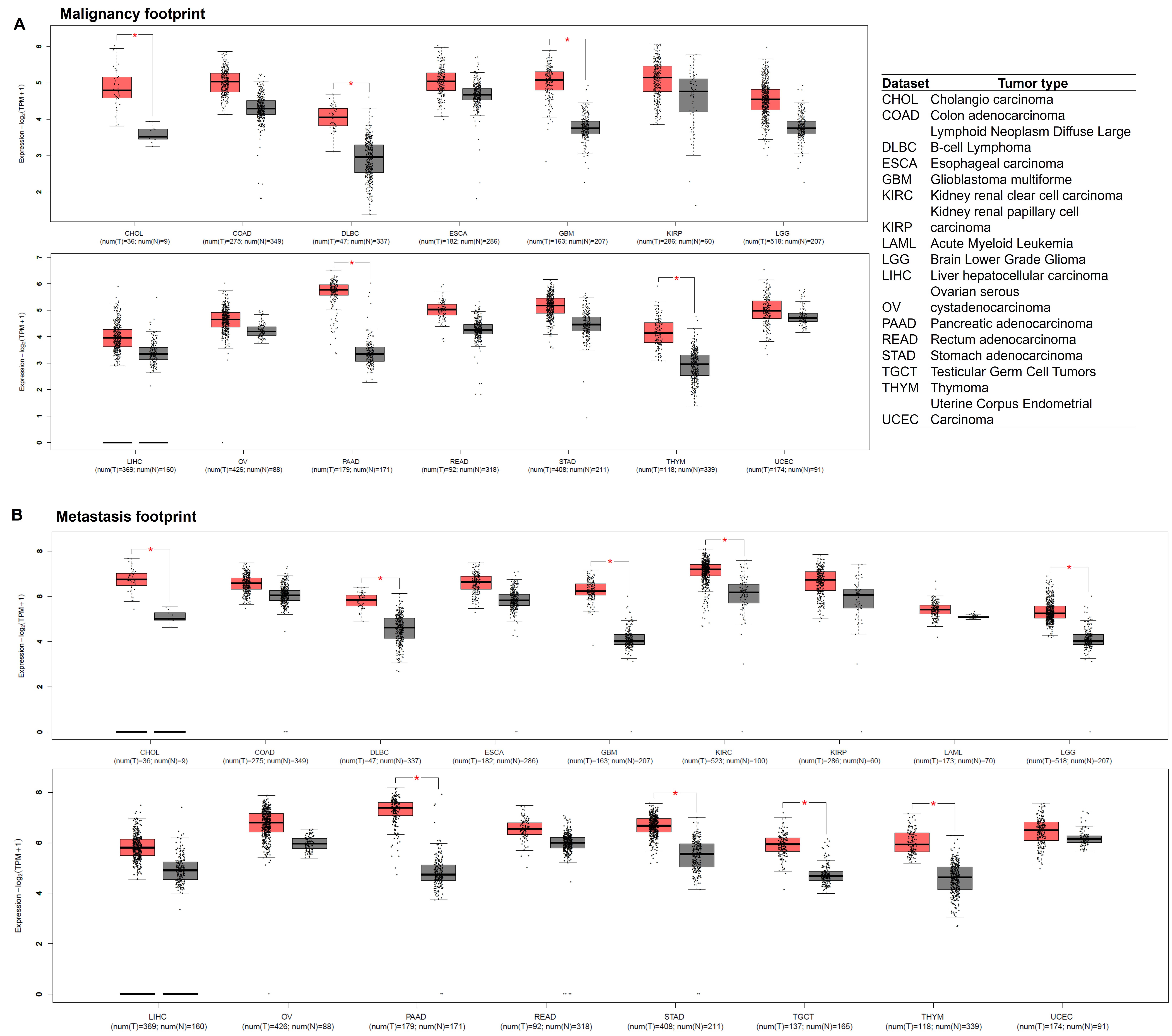
**

**Figure S4. The expression of the curated malignant (A) and metastatic (B) footprints in a variety of cancer datasets from TCGA database.**
